# Dysregulation of Purinergic Signaling Sustains Chronic Inflammation and Oxidative Imbalance in Patients After PitNET Surgical Resection

**DOI:** 10.3390/ijms26146890

**Published:** 2025-07-17

**Authors:** Geile Fistarol, Luiz A. de Oliveira, Gilnei B. da Silva, Daiane Manica, Marceli C. Hanauer, Paula Dallagnol, Rafael A. Narzetti, Maria L. Bergamini, Vitória C. de Melo, Tais Vidal, Micheli M. Pillat, Jussara de Lima, Marcelo L. V. da Cunha, Marielle L. Makiyama, Filomena Marafon, Aniela P. Kempka, Ariane Zamoner, Margarete D. Bagatini

**Affiliations:** 1 Postgraduate Program in Biomedical Science, Federal University of Fronteira Sul, Chapecó 89815-899, SC, Brazil; geile.fistarol@gmail.com (G.F.); albertoufsj@hotmail.com (L.A.d.O.); paula.dallagnol@hotmail.com (P.D.); jhudlima@hotmail.com (J.d.L.); endocrinologistamarielle@yahoo.com (M.L.M.); marafon.filo@gmail.com (F.M.); maria.bergamini@estudante.uffs.edu.br (M.L.B.); vitoriacapelli@hotmail.com (V.C.d.M.); 2 Multicentric Postgraduate Program in Biochemistry and Molecular Biology, State University of Santa Catarina, Lages 88520-000, SC, Brazil; gilnei.silva@edu.udesc.br (G.B.d.S.); aniela.kempka@udesc.br (A.P.K.); 3 Postgraduate Program in Biochemistry, University of Santa Catarina, Florianópolis 88040-970, SC, Brazil; daianemanica2011@hotmail.com (D.M.); rafaelnarzetti@uricer.edu.br (R.A.N.); arianezps@gmail.com (A.Z.); 4 Department of Nursing, Postgraduate Program in Nursing, Federal University of Santa Catarina, Florianópolis 88040-970, SC, Brazil; tilitobinha@gmail.com; 5 Center of Health Sciences, Federal University of Santa Maria, Santa Maria 97105-900, RS, Brazil; tais.vidal@ufsm.br (T.V.); micheli.pillat@ufsm.br (M.M.P.); 6 Regional Hospital of West, Center of Neurosurgery, Chapecó 89812-505, SC, Brazil; marcelolvc@yahoo.com.br

**Keywords:** neuroendocrine tumor, surgical resection, purinergic signaling, ectonucleotidases, inflammation, oxidative stress

## Abstract

Pituitary neuroendocrine tumors (PitNETs) are the most common intracranial tumors. Evidence suggests that these types of tumors may have high recurrence rates. In this context, the purinergic system, oxidative stress, and inflammation are important signaling pathways involved in the cancer’s pathophysiology. This study aimed to evaluate the sociodemographic and diagnostic profiles, as well as assess the purinergic signaling, immunological, and redox profiles, of patients after PitNET resection. We collected sociodemographic data and the patients’ diagnostic profiles. We also collected blood samples to analyze glycemia, triglycerides, albumin, and ATP levels. The ectonucleotidase activity was determined in peripheral blood mononuclear cells (PBMCs). In addition, we evaluated their redox and immunological profiles. There was a prevalence of gonadotropic macroadenoma derived from PIT-1 cells. We found that patients included in the PitNET group had increased glycemia, serum ATP levels, and ATP hydrolysis in PBMCs. Analyzing their immunological profiles, we found that patients had increased levels of IL-6, IL-10, and TNF, while the IL-27 level was decreased. Regarding their redox profiles, PitNET patients had increased levels of ROS and protein carbonylation. Unexpectedly, patients also showed increased levels of non-protein thiols (NPSHs), total thiols (PSHs), and ascorbic acid. Thus, the dysregulation of purinergic signaling sustained chronic inflammation and oxidative imbalance in PitNET patients for a long time after surgical resection. These data suggest that patients with PitNETs require long-term accompanying to prevent cancer recurrence prognosis. The biomarkers highlighted in this study may be good tools to help the medical approaches.

## 1. Introduction

Pituitary neuroendocrine tumors (PitNETs) are malignant masses of cells derived from adenohypophysis that affect the neurologic–endocrine axis [1]. Epidemiologically, these malignant neoplasms have an incidence of 3.9 to 7.4 cases per 100,000 inhabitants per year and a prevalence of 76 to 116 cases per 100,000 inhabitants [2]. In addition, PitNETs are the most common type of disease affecting the pituitary gland, accounting for approximately 15% of all brain tumors [3,4].

The possible treatments for PitNETs include anti-secretory drugs, surgical resection, chemotherapy, radiotherapy, and immunotherapy [5,6]. However, due to the aggressive nature of PitNETs [5], traditional treatments may have no sustainable effects [7], and tumor recurrence in resected patients is a concern that limits life quality [8,9]. In this panorama, the previous retrospective cohort revealed that 45.71% of patients who received surgical management of the tumors had recidivism [10], which indicates that PitNETs have high rates of recurrence.

Nowadays, several studies have shown that purinergic signaling is involved in the cancer’s pathophysiology. This cell signaling pathway has as a key molecule adenosine triphosphate (ATP), which seems to be increased in the tumor microenvironment (TME) [11,12,13]. In this sense, other dysregulations have also been reported, such as in the hydrolytic activity of CD39 and CD73 enzymes [14,15,16]. Furthermore, this cell signaling pathway also plays a role in inflammation [17,18], which justifies the reason for it to be broadly investigated. Despite that, currently, no evidence shows the involvement of purinergic signaling in PitNETs’ pathophysiology or after surgical resection.

Recent evidence has also shown that purinergic signaling and oxidative stress are interconnected pathways [19] and are present in the tumor context [20,21]. In this context, it is known that redox imbalance produces free radicals, such as reactive oxygen species (ROS), which are molecules capable of causing damage to lipid membranes and DNA [22]. Related to PitNETs, the limited literature available suggests a possible involvement of oxidative stress in the pathophysiology of these neoplasms [23,24], and the need for new solid discoveries in this area is urgent.

Therefore, considering the lack of literature, we aim in this article to highlight the sociodemographic characteristics and cancer classification, as well as evaluate the nuances of the purinergic signaling, oxidative stress, and inflammatory profiles, of long-time post-resected PitNET patients. We hypothesize that the patients have dysregulation of purinergic signaling after surgical resection, which directly impacts their immune profiles and oxidative balance. Taken together, this unfavorable panorama may signal cancer recurrence in the PitNET context, requiring more attentive approaches to prevent a bad long-term prognosis.

## 2. Results

### 2.1. Sociodemographic Characteristics of Participants

Table 1 presents the sociodemographic characteristics of the participants involved in this research, including age, gender, and BMI. The mean age of the individuals in the PitNET group was 57.78 years (±13.52), while in the controls, it was 51.88 (±13.62). We verified a predominance of female individuals (64.70%; *n* = 11) in the PitNET group in comparison with males (35.30%; *n* = 6). Regarding body composition, the mean BMI of patients in the PitNET group was 30.70 (± 7.61), and in the control group, it was 28.79 (± 3.50). Analyzing in detail, in the PitNET group, 29.41% (*n* = 5) of individuals were obese, 52.95% (*n* = 9) were overweight, and 17.64% (*n* = 3) were eutrophic. Similarly, in the control group, 40.90% (*n* = 9) of the participants were obese, 31.82% (*n* = 7) were overweight, and 27.28% (*n* = 6) were eutrophic.

Among the main baseline chronic diseases, we found that arterial hypertension was the most prevalent disease in the PitNET group, with 23.53% (*n* = 4) of the total, followed by the association between arterial hypertension + diabetes (type 2) (11.76%; *n* = 2) and arterial hypertension + dyslipidemia (11.76%; *n* = 2). In addition, 23.53% (*n* = 4) of the patients reported not having any type of chronic disease. Other baseline chronic diseases or associations corresponded to 5.88% (*n* = 1) (see more details in Table 1). Regarding bad habits, smoking corresponded to 11.76% (*n* = 2) and alcoholism to 5.88% (*n* = 1), while 82.35% (*n* = 14) of the PitNET patients did not indicate bad habits.

### 2.2. Tumor Classification of Patients

In this study, we compiled the tumor classification of the patients included in the PitNET group (Table 2), considering the medical diagnosis based on the immunohistochemistry, tumor size, and cell lineage. Thus, 94.11% (*n* = 16) of cases were macroadenomas and 5.88% (*n* = 1) were microadenomas. In the classification of histology, 23.52% (*n* = 4) were cases of the gonadotroph subtype, 17.64% (*n* = 3) the somatotroph subtype, 17.64% (*n* = 3) the multihormonal subtype, 11.76% (*n* = 2) the densely granulated corticotroph subtype, and 11.76% (*n* = 2) null cells. The tumors classified as densely granulated somatotrophs, sparsely granulated lactotrophs, and densely granulated lactotrophs each accounted for 5.88% (*n* = 1) of the total cases. Relative to the cell lineages, 35.30% (*n* = 6) were from PIT-1, 29.41% (*n* = 5) were of no distinct cell lineage, 23.52% (*n* = 4) were from SF-1, and 11.76% (*n* = 2) were from T-PIT.

### 2.3. Levels of Biochemical Markers

**Figure 1:**
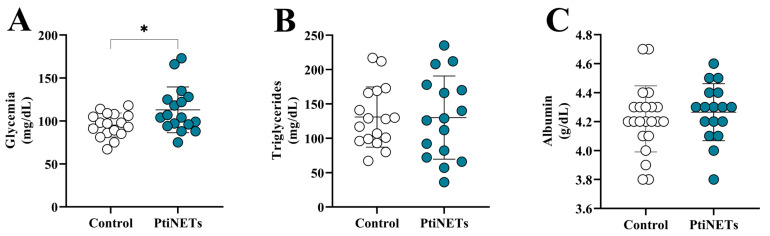
**The levels of biochemical markers.** In comparison with the controls, patients in the PitNET group presented increased glycemia (**A**), while there was no statistical significance for the levels of triglycerides (**B**) and albumin (**C**). Statistical analysis: Student’s *t*-test or the Mann–Whitney test was used to compare the groups. *p* < 0.05 was considered statistically significant. * (*p* < 0.05).

The blood glucose (glycemia), triglyceride, and albumin levels in the PitNET group and controls are shown in Figure 1A–C. Individuals in the PitNET group had increased glucose levels (*p* = 0.0218) when compared with the control group (Figure 1A). However, there was no statistically significant difference for triglyceride (Figure 1B) and albumin (Figure 1C) levels.

### 2.4. Patients Presented Purinergic Signaling Alterations

**Figure 2:**
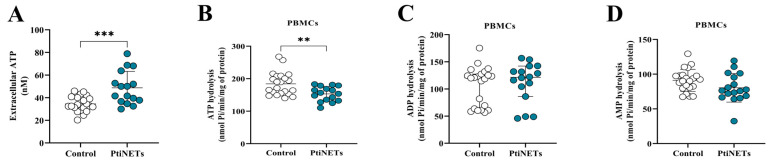
**Extracellular ATP (eATP) levels and ectonucleotidases activity in PBMCs.** We found that the PitNET group had significantly increased levels of ATP in serum (eATP) in comparison with the control group (**A**). Regarding the ectonucleotidase activity, we found an increased hydrolysis of ATP (**B**), while there was no statistical significance for hydrolysis of ADP (**C**) and AMP (**D**). Statistical analysis: Student’s *t*-test or the Mann–Whitney test was used to compare the groups. *p* < 0.05 was considered statistically significant. ** (*p* < 0.01); *** (*p* < 0.001).

Seeking possible alterations in purinergic signaling, we measured the serum eATP levels, as well as the ectonucleotidase activity in the hydrolysis of nucleotides in PBMCs of both groups (Figure 2A–D). Curiously, we found that the PitNET group had expressively increased levels of eATP (*p* = 0.0004) in comparison with the control group (Figure 2A). In addition, we also found that the PitNET group presented significantly decreased levels of ATP hydrolysis (*p* = 0.0039) (Figure 2B), while there were no statistically significant differences between the PitNET and control groups for the hydrolysis of ADP (Figure 2C) and AMP (Figure 2D).

### 2.5. Patients Had Alterations in Cytokine Profiles

**Figure 3:**
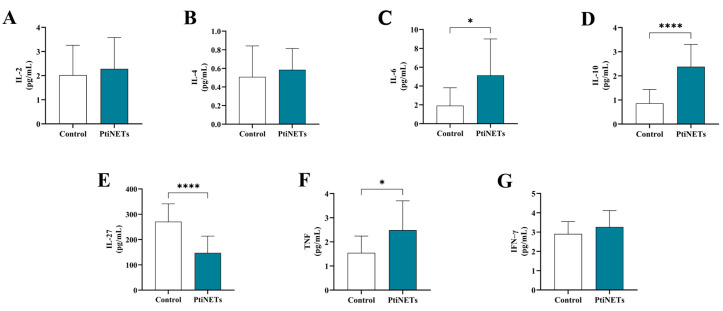
**Immunological profiles.** The levels of IL-2 (**A**), IL-4 (**B**), and IFN-γ (**G**) were unchanged, while IL-6 (**C**), IL-10 (**D**), and TNF (**F**) were increased in patients with PitNETs. In the case of IL-27, levels were reduced in the PitNET group (**E**). Statistical analysis: Student’s *t*-test or the Mann–Whitney test was used to compare the groups. *p* < 0.05 was considered statistically significant. * (*p* < 0.05); **** (*p* < 0.0001).

The results of the dosage of cytokines are shown in Figure 3A–G. The patients belonging to the PitNET group presented increased levels of IL-6 (*p = 0.0413*) when compared with the control group (Figure 3C). Similarly, the levels of IL-10 (*p* < 0.0001) (Figure 3D) and TNF (*p* = 0.0259) (Figure 3F) were also significantly increased. Meanwhile, we found highly reduced levels of IL-27 (*p* < 0.0001) in individuals belonging to the PitNET group when compared with the control group (Figure 3E). There was no statistical significance for the levels of IL-2 (Figure 3A), IL-4 (Figure 3B), and IFN–γ (Figure 3G).

### 2.6. Patients Had an Imbalance in Their Redox Profiles

**Figure 4:**
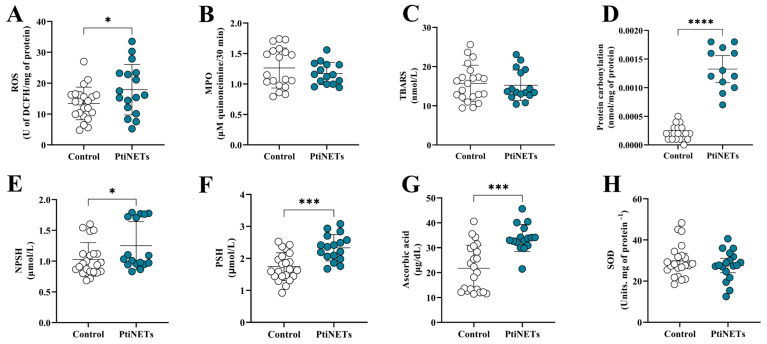
**Redox profiles.** We discovered that patients from the PitNET group had a redox imbalance, with increased levels of ROS (**A**) and protein carbonylation (**D**). In contrast, we also verified an increase in the levels of NPSH (**E**), PSH (**F**), and ascorbic acid (**G**) in the PitNET group. We did not find any statistical significance for the levels of MPO (**B**), TBARS (**C**), and SOD (**H**). Statistical analysis: Student’s *t*-test or the Mann–Whitney test was used to compare the groups. *p* < 0.05 was considered statistically significant. * (*p* < 0.05); *** (*p* < 0.001); **** (*p* < 0.0001).

The redox profiles of the enrolled subjects are shown in Figure 4A–H. The PitNET group had increased levels of ROS (*p* = 0.0466) in comparison with the control group (Figure 4A). Similarly, patients in the PitNET group also presented high levels of protein carbonylation (*p* < 0.0001) (Figure 4D). However, there was no statistical significance for the pro-oxidant marker levels of MPO (Figure 4B) and TBARS (Figure 4C). Regarding the antioxidant panorama, compared with the controls, we found elevated levels of NPSH (*p* = 0.0411) (Figure 4E), PSH (*p* = 0.0001) (Figure 4F), and ascorbic acid (*p* = 0.0001) (Figure 4G). We did not find statistical differences in the levels of SOD in the comparison of the control versus PitNET groups (Figure 4H).

## 3. Discussions

As in other cancer types that also require surgical resection, hypophysectomy with extensive removal of the pituitary mass is the usual treatment for neuroendocrine neoplasms that affect this gland. This approach is considered a standard in the treatment of patients [26]. However, evidence has highlighted that some PitNET subtypes have aggressive behaviors toward conventional therapies [7]. From this panorama, we set out to understand the possible intricate nuances behind the physiology of patients after PitNET long-term surgical resection. Surprisingly, we found that patients presented dysregulation of purinergic signaling, with high extracellular levels of ATP, which culminated in chronic inflammation involving increased IL-6, TNF, and IL-10 levels and decreased IL-27 levels, as well as a pro-oxidative state accompanied by elevated ROS and protein carbonylation. Taken together, these findings may signal possible neoplastic recurrences in the post-resected patients.

We started our research by verifying the sociodemographic data. Thus, in this study, the mean age of the patients in the PitNET group was 57.78 (± 13.52), being very similar to that found in previous work [27]. Furthermore, the BMI of 30.70 (±7.61) for the individuals in the PitNET group represents a risk to the development of cancer [28,29], including pituitary cancer [30]. We also verified that patients belonging to the PitNET group presented arterial hypertension or an association between arterial hypertension and diabetes. It is known that a high body fat mass may increase the risk of type 2 diabetes and heart disease and increase the risk of developing certain types of cancer [31]. 

In the last update of the WHO to classify endocrine and neuroendocrine tumors, pituitary tumor subtypes were organized according to histology and the cell lineage from which they were derived [32]. Among PitNETs, gonadotrophs originating from PIT-1 cells are one of the most common tumor types [33]. Thus, thinking about elucidating the subtypes of diagnosed PitNETs in this study, we compiled them according to size, histology, and cell lineage (Table 2). Corroborating the data from the literature, we found that the most prevalent tumor was classified as a gonadotropic macroadenoma derived from PIT-1 cells.

In the context of cancer, one of the cell signaling pathways involved in pathophysiology is purinergic signaling, which has been shown to play a role in several cancer types, such as melanoma [34], lung cancer [35], and gastric cancer [36]. A key component of this cell signaling, the nucleotide ATP [13], can be found at higher levels in cancer cells to the detriment of healthy cells [37]. ATP can be released from tumoral cells via cell membrane damage or from membrane channels [38]. We expected that after surgical resection, serum ATP levels would be at a basal level. However, we found high ATP levels in patients in the PitNET group (Figure 2A). This peculiar result regarding ATP levels was also demonstrated by Manica et al. [15] in the melanoma cancer context. These data seem to indicate that some cancer types may release proper molecules into the bloodstream to make niches feasible for future neoplastic recurrences.

Outside the cells, the ectonucleotidases CD39 and CD73 are important components of the purinergic system that regulate the extracellular levels of nucleotides, such as ATP, ADP, and AMP, as well as nucleosides [37]. Thus, considering the role of ectonucleotidases in controlling ATP levels, we performed an assay to verify the enzymatic activity of ectonucleotidases in the hydrolysis of ATP, ADP, and AMP. Convergent with high levels of ATP, we discovered a reduction in the ATPase function of purinergic ectoenzymes (Figure 2B), a result that justifies the increased levels of eATP found in patients. Thus, all these findings indicate that patients in the PitNET group had alterations in purinergic signaling with the cascade toward ATP accumulation in the serum.

Intrinsic to the functions played by ATP, it can activate the P2X7 receptor in inflammatory cells and promote the release of inflammatory cytokines, such as IL-6 and TNF [39,40]. Although a long time had passed since the patients’ tumor resection, we investigated whether there was any possibility that high levels of eATP altered their immune profiles. Curiously, we found that the levels of IL-6, IL-10, and TNF were significantly increased, and the IL-27 level was decreased (see Figure 3). Therefore, our results prove that patients were under a chronic inflammatory process due to high levels of eATP for a long time after surgical resection, a condition that may favor carcinogenesis and means poor prognosis in the long term.

Regarding the function of IL-10 in a cancer context, this interleukin may contribute to tumor progression by interrupting inflammation, leading to antitumor immunity. Meanwhile, it recruits and stimulates cytotoxic CD8+ cells in the tumor microenvironment, promoting immunological memory and suppressing the local release of pro-inflammatory cytokines [41]. In a study of advanced renal cell carcinoma, it was found that increased levels of IL-10 are associated with the potential development of metastasis [42]. In the case of colorectal cancer, increased IL-10 levels have also been linked to a negative prognosis [43]. Based on these excerpts from the literature, the high IL-10 levels found in the patients in our study are another point of concern that may indicate cancer recurrence.

As shown previously, we found decreased levels of IL-27 in patients from the PitNET group. IL-27 is considered the newest member of the IL-6/IL-12 interleukin family [44]. The role of this interleukin in the human body has still not been elucidated, but it seems to act as a pleiotropic molecule. A body of evidence suggests it is involved in inflammation and favors tumors, whereas others indicate an anti-inflammatory and anti-cancer effect [45,46,47]. Despite that, Mascanfroni et al. [48] correlated an increase in CD39 expression positively with IL-27, connecting this interleukin to purinergic signaling. Given the results found for IL-27 levels, it is possible to infer that high levels of ATP may be related to the downregulation of CD39 controlled by the lower levels of IL-27, favoring the accumulation of this molecule in the peripheral bloodstream of PitNET patients for a long time after surgical resection.

It was recently demonstrated that PitNET patients are associated with elevated levels of ROS and signs of oxidative damage, which promotes the recruitment of immune cells and may lead to a pre-neoplastic condition [23]. In this sense, it is known that ATP is capable of ROS stimulation via P2X7 receptor agonism [12]. Since increased levels of ATP in patients in the PitNET group were found and evidence about the pro-neoplastic role of ROS, we sought to characterize the redox profiles of the individuals included in this research. Factually, we found that patients had elevated levels of ROS and protein carbonylation (Figure 4). These results corroborate the hypothesis that patients still presented a redox imbalance after tumor surgical resection, favoring oxidative damage and possible neoplastic resurgence.

Although the patients presented increased levels of pro-oxidant molecules, we also verified that they presented increased levels of ascorbic acid and PSH/NPSH. In this sense, ascorbic acid is known as a potent antioxidant substance [49], as well as PSH/NPSH [50,51]. In addition, a curious fact we found in a previous study was that metformin, used to treat type 2 diabetes, was able to raise the levels of both ascorbic acid and thiols in patients and PBMCs [52]. Thus, as individuals mainly in the PitNET groups were continuously using this drug to treat type 2 diabetes, a possible explanation for our results was that metformin improved the levels of these antioxidants, but without any efficient effect on oxidative molecules, since increased ROS and protein carbonylation was detected.

All the results found in this research demonstrate that patients with PitNETs require long-term follow-up due to the consequences of purinergic signaling dysregulation, particularly on oxidative stress and cytokine profiles, which can favor tumor recurrence. Moreover, we encourage further studies to deepen the understanding of the impacts of purinergic signaling in other neuroendocrine tumors and to expand the knowledge and medical care for patients.

## 4. Materials and Methods

### 4.1. Study Design and Participant Selection

This cross-sectional study was conducted throughout January 2024. A total of 39 subjects residing in the municipality of Chapecó, western Santa Catarina state (SC), were included. Of this total, 17 were patients (male and female) selected by the researchers’ prior contact with the neurology service of the Hospital Regional do Oeste (HRO), in Chapecó, SC. The volunteer patients were 18 years old or older and had a previous diagnosis of PitNETs according to the hospital’s neurology/oncology department. In addition, the selected patients had undergone surgical resection of the tumor approximately 12 to 24 months before this research. The patients included in this study were taking medications to treat baseline diseases, including antihypertensives, antidiabetics, antidepressants, HMG-CoA reductase inhibitors, and levothyroxine. Twenty-two healthy individuals without any underlying disease, such as cardiovascular problems, cancer, diabetes, or any other chronic communicable or non-communicable diseases, were also recruited to compose the control group. The control individuals were matched with the patients regarding age and sex.

This project was approved by the Human Research Ethics Committee (HREC) of the Federal University of the Southern Frontier (UFFS), the Chapecó, SC, campus, under protocol number 6.602.966. The collection of information and biological materials was carried out only after the signing of the free and informed consent form (FICF).

### 4.2. Anthropometric Assessment

To record anthropometric data, the participants were weighed and measured wearing light clothing and without shoes. A digital scale with a capacity of 150 kg and a portable stadiometer were used. Body composition was classified based on the body mass index (BMI), following the classification table for adults from the World Health Organization [25], applicable to both sexes.

### 4.3. Collection of Biological Material

A total of 30 mL of whole blood was collected from fasting patients and controls by qualified professionals. A vacuum collection system (Vacutainer^®^, BD Biosciences, San Diego, CA, USA) was used in tubes with separating gel, tubes containing EDTA, and tubes containing sodium citrate. The collected material was homogenized and transported in a polystyrene box containing ice to the processing local.

### 4.4. Processing of Biological Material and Separation of Peripheral Blood Mononuclear Cells (PBMCs)

The collected samples were processed in the biochemistry laboratory of the UFFS, the Chapecó, SC, campus. The blood collected in sodium citrate tubes was stored in microtubes at −80 °C until the analyses were performed. The blood stored in the tubes with separating gel and EDTA was centrifuged at 3500 rpm for 15 min. Afterward, the serum was obtained from the tubes with separating gel, which was also stored in microtubes at −80 °C until the necessary analyses were carried out.

Regarding the tubes with EDTA, the buffy coat from which the peripheral blood mononuclear cells (PBMCs) were obtained was collected, according to the protocol established by Böyum [53], with adaptations. For this purpose, the buffy coat was diluted in saline solution (1:1). Then, the mixture was transferred to a conical tube containing Ficoll-Histopaque and centrifuged at 1800 rpm for 30 min. After centrifugation, the intermediate layer containing the PBMCs was collected. Then, the cells were transferred to a new tube, washed twice with 10 mL of saline solution, and centrifuged again for 5 min at 1500 rpm. When necessary, the cells were washed with 5 mL of a hemolytic buffer to eliminate red blood cell residues, followed by centrifugation for 5 min at 1500 rpm. The resulting PBMCs were stored in microtubes with 600 μL of saline solution and frozen at −80 °C until experiments were performed to evaluate the enzymatic activity of ectonucleotidases.

### 4.5. Analysis of Peripheral Biochemical Markers

We analyzed the levels of blood glucose, triglycerides, and albumin in participants using colorimetric assays with an automated clinical analyzer (Wiener lab., CM 200). All analyses were performed following the manufacturer’s kits (Wiener lab., Rosario, Argentina). The whole blood from subjects was used to detect the levels of blood glucose, while the triglyceride and albumin levels were analyzed in serum. Briefly, we added into each specific analyzer compartment the colorimetric reagent, buffer solution, and samples. Then, approximately 10 µL of the samples was automatically pipetted into an empty well and mixed with the colorimetric reagent and buffer solution. After the incubation time, the colorimetric reaction was read spectrophotometrically by the equipment. The blood glucose and triglyceride results were expressed in milligrams per deciliter (mg/dL). In the case of albumin, the results were expressed in grams per deciliter (g/dL).

### 4.6. Analysis of Purinergic Signaling

#### 4.6.1. Determination of Extracellular ATP (eATP) Levels

For the determination of extracellular ATP (eATP), the Molecular Probes™ ATP Determination Kit (Invitrogen™, Waltham, MA, USA) was used, following the manufacturer’s recommendations. Each reaction content consisted of 1.25 µg/mL of firefly luciferase, 50 µM of D-luciferin, and 1 mM of DTT in 1x reaction buffer. Then, 10 µL of the serum sample was mixed with 90 µL of the reaction content and incubated at 37 °C for 15 min. After the incubation time, the luminescence was measured with a spectrophotometer at a wavelength of 560 nm (Varioskan™ LUX, Thermo Scientific™, Waltham, MA, USA). An ATP standard curve was prepared at concentrations ranging from 1 nM to 1 µM. The results were expressed in nM of extracellular ATP.

#### 4.6.2. Assessment of Enzymatic Activity of Ectonucleotidases

The activity of ectonucleotidases was evaluated in PBMC samples from control individuals and patients. To evaluate the ATPase, ADPase, and AMPase activities, the substrates ATP, ADP, and AMP were used, respectively, with the measurement of inorganic phosphate (Pi) produced as a result of the hydrolytic activity at the end of each reaction. Thus, after protein adjustments, 20 μL of each PBMC sample was added to a reaction mixture of each enzyme and pre-incubated at 37 °C for 10 min. The reaction was initiated by the addition of the specific substrates for each enzyme. After incubation at 37 °C for 70 min, the reactions were stopped by the addition of 150 μL of trichloroacetic acid (TCA; 15%), and the inorganic phosphate released due to hydrolysis was determined using malachite green as a colorimetric reagent. A standard curve was prepared with KH_2_PO_4_. The absorbance was measured at 630 nm, and the results were presented as nmol/Pi/min/mg protein. The results were corrected for non-enzymatic hydrolysis [54,55].

### 4.7. Analysis of Immunological Profile 

#### 4.7.1. Cytokine Determination by Flow Cytometry

The levels of interleukin-2 (IL-2), interleukin-4 (IL-4), interleukin-6 (IL-6), interleukin-10 (IL-10), interferon-γ (IFN-γ), and tumor necrosis factor (TNF) were determined using the BD™ Cytometric Bead Array (CBA) Human Th1/Th2 Cytokine Kit II (catalog Nº. 551809), following the manufacturer’s recommendations. The results were expressed in picograms per mL (pg/mL).

#### 4.7.2. Determination of IL–27 Levels

The levels of IL-27 were assessed by sandwich immunoassays using a human ELISA kit (Invitrogen^®^), following the manufacturer’s instructions. Firstly, the serum was diluted 2x with a diluent buffer. Afterward, 100 µL of each sample was added to a 96-well microplate, covered, and incubated for 2.5 h at 37 °C with gentle shaking. Then, the supernatant was discarded, and the wells were washed 4x with a wash buffer solution and dried by the inversion of the plate against an absorbent sheet. In the sequence, 100 µL of a biotin conjugate was added to each well and incubated with gentle shaking for 1 h at 37 °C. After the plate was washed 4×, 100 µL of a streptavidin–HRP solution was added, followed by incubation for 45 min at room temperature. Finally, 100 µL of a TMB substrate was added to the wells, and the mixtures were incubated for 30 min at room temperature in the dark. The reaction was stopped with 100 µL of a stop solution. The readings were taken at 450 nm with a spectrophotometer (Varioskan™ LUX, Thermo Scientific™). The results were calculated considering the interpolation of the equation of the absorbance curve by the concentration and were expressed in picograms per milligram of protein (pg/mg).

### 4.8. Analysis of Redox Profile

#### 4.8.1. Reactive Oxygen Species (ROS) 

The level of ROS was estimated by the fluorometric protocol established by Ali et al. [56]. For this, 10 µL of serum was incubated with 10 µL of 2′,7′-dichlorofluorescein diacetate (DCFH-DA; 7 μM) and 240 µL of phosphate-buffered saline (PBS). After 30 min of incubation at 37 °C, the final product of DCFH-DA oxidation, dichlorofluorescein (DCF), was measured. The fluorescence intensity was read with an excitation of 488 nm and an emission of 525 nm (Thermo Scientific™ Varioskan™ LUX). The results were expressed as a percentage (%) of the fluorescence intensity compared to the control.

#### 4.8.2. Thiobarbituric Acid Reactive Substances (TBARS) Assay

We performed a colorimetric assay to analyze lipoperoxidation by measuring the levels of thiobarbituric acid reactive substances (TBARSs). For this assay, we followed the protocol developed by Jentzsch et al. [57], with some modifications. Thus, 20 µL of each serum sample was mixed with 55 µL of distilled water, 100 µL of orthophosphoric acid (0.2 M), and 25 µL of TBA (0.1 M), and the mixture was incubated at 37 °C for 45 min. Finally, the pink product was read with a spectrophotometer (Varioskan™ LUX, Thermo Scientific™) at 532 nm. The results were expressed in nmol/L of TBARS.

#### 4.8.3. Myeloperoxidase (MPO) Enzymatic Activity 

We analyzed the MPO activity in PBMCs from patients and controls according to a study by Suzuki et al. [58]. For this, we used a modified peroxidase system, mixing 12 µL of the samples with 148 µL of amino antipyrine (AAP) in a phenol solution (2.5 mM AAP; 20 mM phenol) and 17 µL of H_2_O_2_ solution (17 mM). After 30 min of incubation at 37 °C, the system was read spectrophotometrically at 492 nm (Varioskan™ LUX, Thermo Scientific™). The results were expressed in μM of quinoneimine per milligram of protein produced in 30 min (μMq/mg/30 min).

#### 4.8.4. Protein Carbonylation 

Serum protein carbonylation was determined by the modified method of Wehr and Levini [59]. First, the proteins from 1000 µL of serum were precipitated with 500 µL of 10% TCA and centrifuged at 5000 rpm for 5 min, discarding the supernatant. Then, the proteins were incubated for 30 min at room temperature with 150 µL of DNPH (10 mM). After incubation for 30 min at room temperature, 500 µL of TCA (10%) was added to the precipitated proteins, and they were centrifuged at 5000 rpm for 5 min. After discarding the supernatant, the red precipitate was washed twice with 1000 µL of ethanol/ethyl acetate (1/1), followed by centrifugation at 10,000 rpm. Subsequently, the supernatant was removed, and the precipitate was dissolved in 1500 µL of a protein denaturation solution (SDS 2%; pH 8.0). Readings were performed with a spectrophotometer at a wavelength of 380 nm. The results were expressed in nM of protein carbonyl per mg of protein.

#### 4.8.5. Ascorbic Acid (Vitamin C)

The levels of ascorbic acid were assessed by the method of Jacques-Silva et al. [60], with adaptations. Briefly, 200 μL of each serum sample was first deproteinized with the addition of an equal volume of trichloroacetic acid (TCA; 10%). Afterward, 100 μL of the remaining supernatant was mixed with 25 μL of distilled water, 25 μL of TCA (13.3%), and 20 μL of 2,4-dinitrophenylhydrazine (DNPH), followed by 2 h of incubation at 37 °C. After time elapsed, the reaction was stopped by adding 135 μL of sulfuric acid (65%), and the orange–red product generated was spectrophotometrically read with the absorbance set at 520 nm (Varioskan™ LUX, Thermo Scientific™). The results were expressed in µg/dL.

#### 4.8.6. Total Thiol (PSH) and Non-Protein Thiol (NPSH)

Both levels of thiols were determined according to Ellman [61], with adjustments. For the total thiol assay, 30 µL of serum in a 96-well plate was added to 200 µL of potassium phosphate buffer (PPB) (1M; pH 6.8) and 20 µL of 5,5ʹ-dithiobis (2-nitrobenzoic acid) (DTNB) with immediate reading. For the non-protein thiols, the serum samples were previously deproteinized by adding an equal sample volume of TCA (10%), and the remaining supernatant was used. Then, 40 µL of each sample was mixed with 260 µL of PPB and 15 µL of DTNB, with immediate reading. All reads were measured at 412 nm (Varioskan™ LUX, Thermo Scientific™). The results were expressed in µM, using a cysteine standard curve as the parameter.

#### 4.8.7. Superoxide Dismutase (SOD) Enzymatic Activity

The superoxide dismutase (SOD) enzyme activity was determined in whole-blood samples from patients and controls, according to the study by McCord and Fridovich [62]. Initially, the concentrated blood samples were diluted in PBS (pH 7.2). Afterward, the samples were mixed with 180 µL of a glycine buffer (50 mM; pH 10.5). The reaction was initiated by adding 10 µL of an adrenaline solution (60 mM; pH 2.0), followed by kinetic readings every 30 s, for a total of 10 min, at a wavelength of 480 nm (Varioskan™ LUX, Thermo Scientific™). Adrenaline’s auto-oxidation was used to correct the values obtained in the samples. The results were expressed in units per milligram of protein^−1^ (units.mg of protein^−1^).

### 4.9. Protein Dosage of Samples 

The quantification of the protein levels in the samples was determined according to the Bradford [63] method and adjusted as required for each analysis. When necessary, the samples were diluted with saline solution or as required by the protocols.

### 4.10. Statistical Analysis 

Statistical analysis was performed using the GraphPad Prism 9.0.1 software (GraphPad Software, San Diego, CA, USA). The data normality was analyzed using the Shapiro–Wilk test. Outliers were analyzed and removed using the Grubbs test. Regarding the studied variables, differences between groups were evaluated using Student’s *t*-test for parametric data and the Mann–Whitney test for non-parametric data. The results were presented as means ± standard deviations for parametric variables and as medians and 95% confidence intervals (95% CIs) for non-parametric variables. Differences for which the probability of rejecting the null hypothesis was less than 5% (*p* < 0.05) were considered statistically significant.

## 5. Conclusions

For the first time, we found that after surgery resection of PitNETs, patients present dysregulation of purinergic signaling, with high extracellular levels of ATP, which culminates in chronic inflammation and a pro-oxidant state. Taken together, these findings bring to light the possibility of patients having tumor relapses throughout their lives due to poor prognosis markers left by PitNETs. In addition, the biomarkers highlighted in this study may be good tools to help medical approaches. We further highlight that few studies in the literature have focused on broadly understanding this tumor type, and that is why we suggest new prospective longitudinal studies be developed, carrying out post-surgical monitoring of these patients to verify the recurrence rate.

## Figures and Tables

**Table 1:** Sociodemographic characterization of subjects. * Based on WHO [25] parameters.

	Subject Groups	
**Characteristics**	**PitNETs** *n* = 17	**Controls** *n* = 22
**Age (years old)**	57.78 ± 13.52	51.88 ± 13.62
**Gender (%)**		
Male	35.30 (*n* = 6)	*50.00* (*n* = 11)
Female	64.70 (*n* = 11)	*50.00* (*n* = 11)
**BMI** (kg/m^2^) *	30.70 ± 7.61	28.79 ± 3.50
Eutrophic	17.64 (*n* = 3)	27.28 (*n* = 6)
Overweight	52.95 (*n* = 9)	31.82 (*n* = 7)
Obesity	29.41 (*n* = 5)	40.90 (*n* = 9)
**Baseline chronic diseases (%)**		
Arterial hypertension	23.53 (*n* = 4)	-
Diabetes (type 2)	5.88 (*n* = 1)	-
Hypothyroidism	5.88 (*n* = 1)	-
Dyslipidemia	5.88 (*n* = 1)	-
Arterial hypertension + diabetes (type 2)	11.76 (*n* = 2)	-
Arterial hypertension + dyslipidemia	11.76 *(n* = 2)	-
Hypothyroidism + depression	5.88 (*n* = 1)	-
Arterial hypertension + diabetes (type 2) + dyslipidemia + depression	5.88 (*n* = 1)	
No disease	23.53 (*n* = 4)	-
**Bad habits (%)**		
Smoking	11.76 (*n* = 2)	-
Alcoholism	5.88 (*n* = 1)	-
No bad habits	82.35 (*n* = 14)	-

**Table 2:** Tumoral classification according to size, hormone production, and cell lineage.

Tumor Classification	
**By size**	
Microadenoma	5.88% (*n* = 1)
Macroadenoma	94.11% (*n* = 16)
**By histological subtype**	
Densely granulated somatotroph	5.88% (*n* = 1)
Somatotroph	17.64% (*n* = 3)
Sparsely granulated lactotroph	5.88% (*n* = 1)
Densely granulated lactotroph	5.88% (*n* = 1)
Densely granulated corticotroph	11.76% (*n* = 2)
Gonadotroph	23.52% (*n* = 4)
Multihormonal	17.64% (*n* = 3)
Null cell	11.76% (*n* = 2)
**By cell lineage**	
PIT-1	35.30% (*n* = 6)
T-PIT	11.76% (*n* = 2)
SF-1	23.52% (*n* = 4)
No distinct cell lineage	29.41% (*n* = 5)

## Data Availability

The datasets generated during and/or analyzed during the current study are not publicly available but are available from the corresponding author upon reasonable request.
